# Assessment of brain atrophy as a promising marker of radiological activity in patients with relapsing–remitting multiple sclerosis

**DOI:** 10.3389/fnins.2025.1661539

**Published:** 2025-10-07

**Authors:** Aleksandra Pogoda-Wesołowska, Ignacy Stachura, Piotr Szukało, Maria Wieczorek, Adam Stępień

**Affiliations:** ^1^Neurology Clinic, Military Institute of Medicine – National Research Institute, Warsaw, Poland; ^2^Faculty of Physics, University of Warsaw, Warsaw, Poland; ^3^Faculty of Medicine, Medical University of Warsaw, Warsaw, Poland

**Keywords:** multiple sclerosis, relapses, lesions, no evidence of disease activity, atrophy, correlation

## Abstract

**Introduction:**

The measurement of brain atrophy in patients with relapsing–remitting multiple sclerosis (RRMS) may be a marker of the disease activity. However, currently this method is not widely used in clinical practice. In the presented study, the relationship between lesions (T2) in magnetic resonance imaging (MRI), including contrast-enhancing (Gd+), clinical relapses and no evidence of disease activity (NEDA-3) with volumetric changes was investigated.

**Methods:**

Clinical and MRI data from RRMS patients treated with cladribine tablets (CLAD) and alemtuzumab (ALEM) were retrospectively analyzed at 4 time points (pretreatment and 3 years of follow-up). Volumetric data were obtained using the FreeSurfer. Annual volumetric changes and new T2/Gd + lesions were pooled together to assess short-term relationships, baseline T2/Gd + lesions were correlated with 3-year volume changes and years with NEDA-3 and without NEDA-3 were compared.

**Results:**

The study included 33 patients treated with CLAD and 19 patients treated with ALEM. In the year-to-year analysis (n_CLAD_ = 59, n_ALEM_ = 36) within the CLAD group, new T2 lesions were significantly associated with a decrease in thalamic (*p* = 0.02), cerebellum (*p* = 0.05) and deep grey matter (*p* = 0.05) volume. When analyzing the correlation between baseline T2 lesions and overall 3-year volume changes (N_CLAD_ = 9, N_ALEM_ = 7), in the CLAD group, strong associations were found with whole brain (*p* = 0.001, *ꞵ* = −0.89), cerebellum (*p* = 0.002, *ꞵ* = −0.20), cerebellar cortex (*p* = 0.003, *ꞵ* = −0.19) and DGM (*p* = 0.015, *ꞵ* = −0.04) atrophy, as well as with lateral ventricular volume increase (*p* = 0.00001, *ꞵ* = 0.1). A similar situation occurred when only the first year of treatment was analyzed (N_CLAD_ = 29, N_ALEM_ = 13). It was not observed in the ALEM group. Interestingly, no correlation was noted between Gd + lesions and volumetric changes. Remarkably, no statistically significant differences between years with and without relapses were observed. However, years without NEDA-3 (*n* = 31) were characterized by greater atrophy in white matter (*p* = 0.04), thalamus (*p* = 0.02), and putamen (*p* = 0.04).

**Conclusion:**

The results of the presented study suggested an association of increased brain atrophy with radiological activity rather than with relapsing disease activity. However, further studies with larger numbers of patients are needed to verify these associations more precisely.

## 1 Introduction

Multiple sclerosis (MS) is a chronic, neuroinflammatory disease of the central nervous system (CNS) that predominantly affects young adults. It is characterized by the immune system’s attack resulting in damage to the myelin sheath, axonal loss and the formation of lesions with subsequent neurodegeneration (Rodríguez Murúa et al., 2022). In MS, inflammation and neurodegeneration are now understood to occur together, and both contribute to the progression of disability.

Magnetic Resonance Imaging (MRI) is the cornerstone of MS diagnosis and monitoring, owing to its high sensitivity in detecting demyelinating lesions. It plays a key role in assessing dissemination in space (DIS) and dissemination in time (DIT) according to the 2017 McDonald criteria, facilitating earlier and more accurate diagnosis by revealing characteristic lesion patterns and their temporal progression (Thompson et al., 2018). MRI-based outcomes contain measurements such as volume and number of T2 hyperintense lesions, including contrast-enhancing (Gd+), and assessment of lesion activity based on the number of new and enlarging lesions. Lesions can also be further classified into specific subtypes, such as paramagnetic rim lesions (PRLs) and those exhibiting the central vein sign (CVS), both of which provide additional insight into disease pathology, and are being studied as possible imaging markers of the disease (Filippi et al., 2019; Preziosa et al., 2021). Moreover, quantitative MRI allows for the evaluation of brain atrophy, a marker of neurodegeneration in MS (Granziera et al., 2021).

Current disease-modifying therapies (DMTs) for MS aim to alter the natural history of the disease by reducing relapse rates and minimizing radiological disease activity. The therapeutic goal is often framed in terms of achieving NEDA-3 (No Evidence of Disease Activity-3), which comprises three key criteria: absence of clinical relapses, no new or enlarging lesions on MRI, and no sustained disability progression as measured by clinical scales such as the Expanded Disability Status Scale (EDSS; Prosperini et al., 2021). The NEDA-3 index is used to assess the response to DMTs, supporting further clinical decision-making. Recently, an increasingly widely discussed NEDA-4 index incorporates additionally brain volume loss (BVL), providing a more sensitive assessment of disease progression. While relapses and MRI-detected lesion activity are indicators of inflammatory processes, whole-brain atrophy represents the result of neurodegeneration and is associated with physical disability (Andravizou et al., 2019). However, the implementation of NEDA-4 in clinical practice is currently restrained by logistical and technical difficulties.

Despite significant progress in the clinical treatment of MS patients, the mechanisms driving the accumulation of disability are not fully understood. One of the described phenomena causing the accumulation of disability are neuroinflammatory events occurring in clinical relapses (relapse-associated worsening—RAW; Chen et al., 2022). To assess the effectiveness of treatment and disease progression, among others, the annual relapse rate (ARR) is used, which represents the average number of relapses experienced by a group of patients per year.

Although a recent systematic review about relationship of disability progression with BVL indicated significant heterogeneity across studies regarding study population, BVL definition, and methodologies for image analysis and clinical measures, whole BLV, increased ventricular volume, and atrophy of grey matter (GM) and corpus callosum were found to be associated with greater disease progression in patients with MS (Matthews et al., 2023). Furthermore, most existing studies suggested no correlation between white matter (WM) atrophy and physical disability in MS. Interestingly, it was noted that it is difficult to establish an association between basal ganglia volume loss and medulla oblongata width and functions impairment in MS patients due to very limited data.

In addition, in the study by Lomer et al., several prognostic factors were associated with MS progression, including the presence of cortical lesions, GM volume changes, BVL, thalamic volume changes, and cross-sectional abnormalities of the spinal cord (Lomer et al., 2024). For cognitive impairment in MS patients, cortical GM volume, BVL, demyelinating lesions characteristics (T2-weighted lesions burden, temporal, frontal, and cerebellar lesions), WM lesions volume, thalamic volume, and corpus callosum density were reliable predictors. This suggests that MRI can be used to predict cognitive decline, disability progression, and disease progression in MS patients over time.

As can be seen, despite the increasingly common use of brain atrophy assessment in MRI to predict disability progression in relapsing–remitting MS (RRMS) patients, the role of this marker in determining its relationship with relapse and radiological activity of the disease still remains to be fully explored and established. Moreover, although volumetric brain assessment is not currently widely used in clinical practice, it may become an important indicator in assessing the effectiveness of DMTs and predicting the further course of the disease. The impact of volumetric changes in specific brain regions on disability progression, clinical relapses, and radiological activity remains insufficiently defined, particularly in patients undergoing highly effective therapies such as Immune Reconstitution Therapies (IRT).

IRT as a highly effective treatment for RRMS includes partially selective therapies, such as cladribine tablets (CLAD), and non-selective therapies, including alemtuzumab (ALEM). ALEM and CLAD are two high-efficacy treatments given as discrete courses separated by 1 year, followed by a durable clinical response that potentially does not require ongoing treatment (Bose et al., 2021). These therapies are now known to be effective in controlling disease relapses and the progression of clinical disability, as demonstrated by both clinical trials and real-world evidence (RWE; Steingo et al., 2020; Stępień et al., 2023; Ziemssen et al., 2020; Giovannoni et al., 2010; Costa-Frossard França et al., 2024; Pogoda-Wesołowska et al., 2025). However, data assessing the effect of IRT on the atrophy process in MS patients are lacking, and the available reports mainly assess the volume loss of the whole brain, without dividing it into individual brain regions (atrophy pattern; Coles et al., 2017; Havrdova et al., 2017; Traboulsee et al., 2016; Filippi et al., 2000; De Stefano et al., 2018; Cortese et al., 2023).

In this study, the relationship between clinical and radiological activity and brain volumetric changes in patients with RRMS treated with IRT - CLAD and ALEM was assessed. The relationship between T2 and Gd + lesions and brain atrophy was measured, and the influence of relapse activity as well as the achievement of NEDA-3 on the volume loss was also examined.

## 2 Methods

### 2.1 Participants

A retrospective, observational, longitudinal study was conducted at the Neurology Clinic of the Military Institute of Medicine—National Research Institute (MIM-NRI). Due to the fact that this was a retrospective study, no restrictive inclusion and exclusion criteria were established and no patient enrollment timeline was used. All patients with RRMS diagnosed according to the 2017 McDonald criteria by neurologist specialists, who had ever been treated with CLAD or ALEM were recruited to the study. The inclusion criteria were the following: patients with RRMS, age over 18, receiving CLAD or ALEM treatment. The exclusion criteria were: patients with other forms of MS, patients under 18 years of age, patients taking DMTs other than IRT in time of analysis, the presence of cancer, pregnancy and breastfeeding.

Demographic data (age, gender, disease duration, number of previous therapies) and clinical data (ARR, EDSS score, number of new T2 lesions, including Gd+) were collected, as well as MRI examinations at four time points: before treatment, 1 year after treatment, 2 years after treatment, and 3 years after treatment. Prior to statistical analysis, all the clinical, demographic and MRI data of patients were stripped of ID (number assigned by the hospital for identification in the medical records system) and PESEL number (unique identification number assigned to Polish citizens and residents, used for personal identification in official records). Patients were marked with acronyms. This was a retrospective study and written consent from participants was not required. However, the project application was sent to the MIM-NRI Bioethics Committee. Resolution No. 54/24 (dated October 16, 2024) was issued, according to which the study was not subject to review by the Bioethics Committee.

### 2.2 Definitions

NEDA—3 was defined as no clinical relapses, no confirmed EDSS disability progression sustained for at least 6 months (if baseline EDSS 0, EDSS increase <1.5 points; if baseline EDSS > = 1, EDSS increase <1 point; if baseline EDSS >5, EDSS increase <0.5 points), no new Gd + lesions, no new or newly enlarging T2 lesions (Beadnall et al., 2019).

### 2.3 MRI examination

All patients were scanned using the same MRI operating system on a General Electric Discovery MR750W3T 3.0 Tesla in the MIM-NRI Magnetic Resonance Imaging Laboratory. A standard protocol for MS was used, including sagittalis (sag) and axialis (ax) T2-weighted gradient-echo (T2) PROPELLER sequence, ax fluid-attenuated inversion recovery sequence (FLAIR), sag CUBE FLAIR sequence, ax diffusion-weighted imaging (DWI) sequence, three- dimensional (3D) susceptibility-weighted angiography (SWAN) sequence and ax 3D T1 pre- and post-contrast sequences. Baseline MRI examinations were performed before the initiation of therapy. As part of routine follow-up of new T2 lesions (including Gd + lesions), yearly control MRI examinations were performed. Not all patients had MRI examinations performed at all four time points, mainly due to the fact that they had not yet completed 3 years of follow-up at the time of analysis. Some MRI examinations were not available for reasons beyond the investigators’ control (they were not performed at all, they were performed in another center). MRI data were acquired from the hospital Alteris system as DICOM file folders in 0.6 mm axial 3D T1 and anonymized.

### 2.4 Brain segmentation

MRI examinations were analyzed volumetrically using Freesurfer software (version 7.4.0). Segmentation of brain structures based on each subject 3D T1-weighted MRI was performed automatically for each patient using automated longitudinal FreeSurfer processing pipeline (Fischl et al., 2002; Fischl et al., 2004). No manual editing was performed to keep methods as automated as possible, and scans with segmentation errors/failures were excluded. Volumetric measurements were also verified by two qualified neurologists. MRI examinations of poor quality (unable to analyze by Freesurfer software due to, for example, artifacts, different MRI protocol) (2 scans) and performed less than 8 weeks after intravenous steroid administration (2 scans) were excluded from the analysis. Assessment of the progression of brain atrophy was based on the comparison of volumes of different structures at different timepoints of the treatment period.

### 2.5 Statistical analysis

All regional volumes were extracted from the FreeSurfer longitudinal pipeline and normalized to each subject’s estimated total intracranial volume (eTIV)—which remains constant across all timepoints—yielding unitless percentages of eTIV. For every ROI, absolute change in volume between yearly separated visits and corresponding number of new T2/Gd + lesions at the beginning of the year were calculated.

We assessed correlations, testing whether the associations between new T2 and Gd + lesions and volume changes differed between the CLAD and ALEM groups, using two approaches:

- **Pooled “patient-year” analysis**

Annual volume changes and the corresponding number of new lesions from the same year were pooled across all patients and years, resulting in 95 observations, which we refer to as patient-years. A patient-year represents the annual volume change together with the number of new lesions for that year; thus, a patient with scans available at all four consecutive time points contributes three patient-year observations. We believe this approach captures short-term associations between new T2/Gd + lesions and volumetric changes.

To account for repeated observations from the same subjects, a linear mixed-effects model was used. In this model, the annual volume change was regressed on the number of new lesions, treatment group (ALEM/CLAD), and their interaction, with a random intercept for each patient, according to the following equation:

$$\Delta V_{ij}=\beta_{0}+\beta_{1}\;T2_{ij}+\beta_{2}\;\mathrm{grou}p_{i}+\beta_{3}\;\left(T2_{ij}\times \mathrm{grou}p_{i}\right)+b_{0i}+\in_{ij}$$

where $\Delta V_{ij}$ represents the volume change for patient i at year j, $\beta_{0}$ represents the intercept, $\beta_{1}$ , $\beta_{2}$ , $\beta_{3}$ are fixed-effect coefficients for lesions, group, and their interaction, $b_{0i}$ is the patient-specific random intercept, and $\in_{ij}$ is the residual error. In the Results section, we focus on differences between the two groups and therefore report *p*-values corresponding to the interaction term.

- **Within-subject longitudinal analysis**

For each individual, volume change over three-year (n_CLAD_ = 9, n_ALEM_ = 7; for this analysis MRI scans from time point 0 and time point 3 were used) and one-year (n_CLAD_ = 29, n_ALEM_ = 13; for this analysis time point 0 and time point 1 were used) intervals was regressed against the corresponding number of new T2/Gd + lesions at baseline.

Two-sided *p*-values were reported for all analyses.

To compare structural atrophy patterns between periods of stable disease and active disease, all patient-years were classified according to the presence or absence of disease activity, as defined by the NEDA-3 criteria. Each interval was categorized as either with NEDA-3 or without NEDA-3.

Percentage volume change for each brain structure was calculated as: (volume_follow-up–volume_baseline)/volume_baseline × 100%. These percentage volume changes were then compared between the with NEDA-3 and without NEDA-3 using Mann–Whitney U test, two-sided *p*-values were reported. In the same way, years with and without relapses were compared.

This study did not include a predefined hypothesis regarding an overarching correlation between brain volume changes and T2/Gd + lesion activity. Instead, the analysis was exploratory, focusing on individual brain structures and treating each structure-specific correlation as an independent analysis. Accordingly, no correction for multiple comparisons was applied, as the goal was not to control the experiment-wise error rate but to generate hypotheses and identify potentially meaningful patterns (Althouse, 2016). Therefore, the reported *p*-values should be interpreted as descriptive and hypothesis-generating, rather than confirmatory.

## 3 Results

### 3.1 Baseline characteristic of the groups

The study initially included 33 patients treated with CLAD (mean age 37.6 years; mean disease duration 8.77 years; 73% women, median baseline EDSS 3; mean baseline T2/Gd + lesions 3.39/1.27; mean baseline ARR 1.30) and 19 patients treated with ALEM (mean age 33.3 years; mean disease duration 7.19 years; 100% women, median baseline EDSS 4.0; mean baseline T2/Gd + lesions 6.26/2.89; mean baseline ARR 2.26). The initial comparison of groups was presented in Table 1. As can be seen, at baseline the treatment groups were similar in terms of age, duration of disease and number of previous DMTs. In the CLAD group, most patients had previously been treated with dimethyl fumarate (55%), glatiramer acetate (9%), ocrelizumab (9%) and fingolimod (9%), similarly to the ALEM group—dimethyl fumarate 26%, fingolimod 21%, natalizumab (16%) and interferon beta-1a 16%. However, groups differed in terms of ARR and disability assessment on the EDSS scale—patients treated with ALEM were characterized by higher disease activity before the start of treatment in terms of relapses and number of T2 and had higher median baseline EDSS score.

**Table 1:** The initial comparison of the treatment groups.

	CLAD (*N* = 33)	ALEM (*N* = 19)	*p*-value
Age of onset	37.7	33.3	0.160
Disease duration	8.77	7.19	0.267
Previous DMTS	2.24	2.78	0.161
Relapses	1.30	2.26	0.002
EDSS score	3.05	3.97	0.034
t2 lesions	3.39	6.26	0.010
GD + lesions	1.27	2.89	0.309

CLAD, cladribine; ALEM, alemtuzumab; DMTs, disease modifying therapies; T2 lesions, new lesions on T2-weighted magnetic resonance imaging scans; Gd + lesions, gadolinium enhancing lesions. Values represent group means. *p*-values were calculated using Student’s t-test or Wilcoxon rank-sum test, as appropriate. *N* = number of patients. The green background color was used to highlight statistically significant values.

### 3.2 Clinical changes within groups in subsequent years of treatment

In the group treated with CLAD, the proportion of patients experiencing relapses dropped from 84.8% before treatment to 24.2% after the first year of therapy, 12.5% after the second year of observation, and reached 46.2% by the third year of follow-up. The average number of new T2 and Gd + lesions decreased from 3.39 and 1.27 before treatment to 0.56 and 0.22 after 1 year of observation, reached 0 for both after 2 years of follow-up, and increased slightly to 0.85 and 0.23 after 3 years of therapy. The median EDSS score was 3.0 prior to treatment, remained stable after the first and second year of therapy, and increased to 3.5 after 3 years of observation.

In the ALEM group, the percentage of patients with relapses declined from 89.5% before treatment to 21% after 1 year of follow-up, 11.1% after 2 years of therapy, and was 23% at the three-year mark. The mean number of new T2 and Gd + lesions prior to therapy was 6.26 and 2.89, dropping to 0.26 and 0.11 after 1 year of treatment, and to 0.17 and 0.11 after 2 years of observation. After 3 years of follow-up, these values increased to 0.92 and 0.5, respectively. The median EDSS score was 4.0 before treatment, decreased to 3.0 after 1 year of follow-up, and remained unchanged during the second and third years of observation.

### 3.3 Year-to-year correlations between new T2/Gd + lesions and volume changes

**Figure 1:**
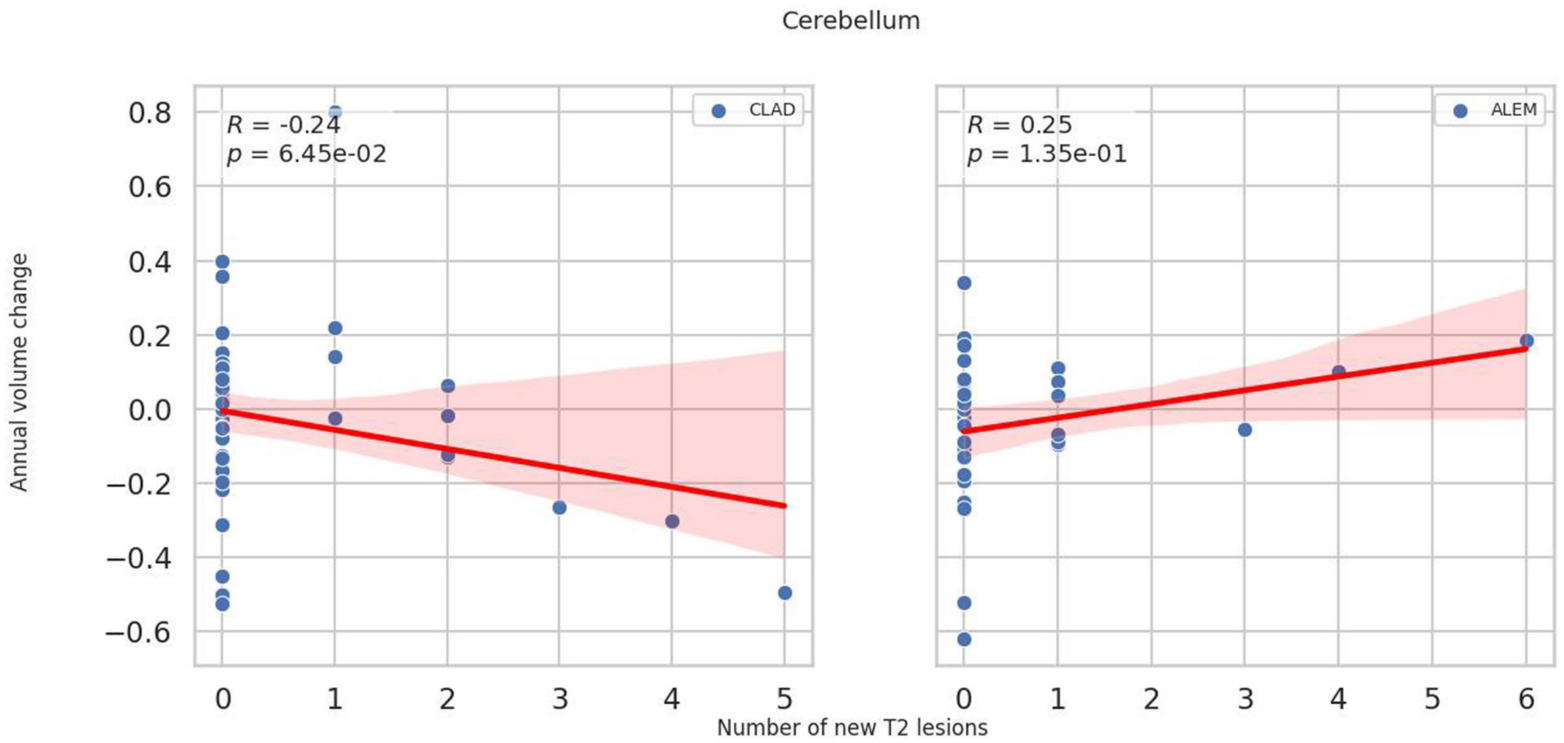
Correlation between the number of new T2 lesions and annual change in cerebellar volume in the corresponding year. Each point represents a patient-year, pooled across the 3-year follow-up. A linear regression line with a 95% confidence interval is shown. The reported *p*-value and R^2^ refer to this simple linear regression and not to the linear mixed-effects model used in the main analysis.

**Figure 2:**
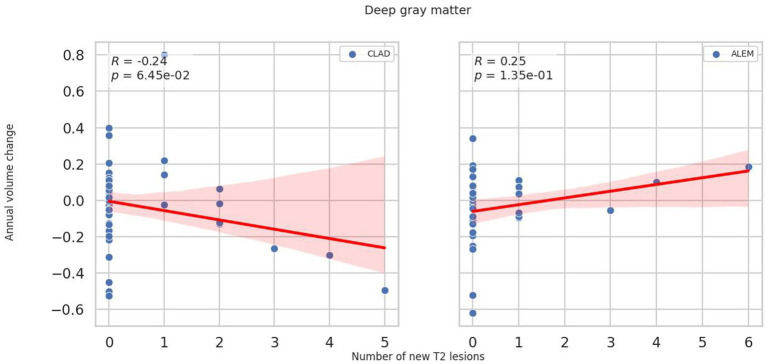
Correlation between the number of new T2 lesions and annual change in deep grey matter volume in the corresponding year. Each point represents a patient-year, pooled across the 3-year follow-up. A linear regression line with a 95% confidence interval is shown. The reported p-value and R^2^ refer to this simple linear regression and not to the linear mixed-effects model used in the main analysis.

**Figure 3:**
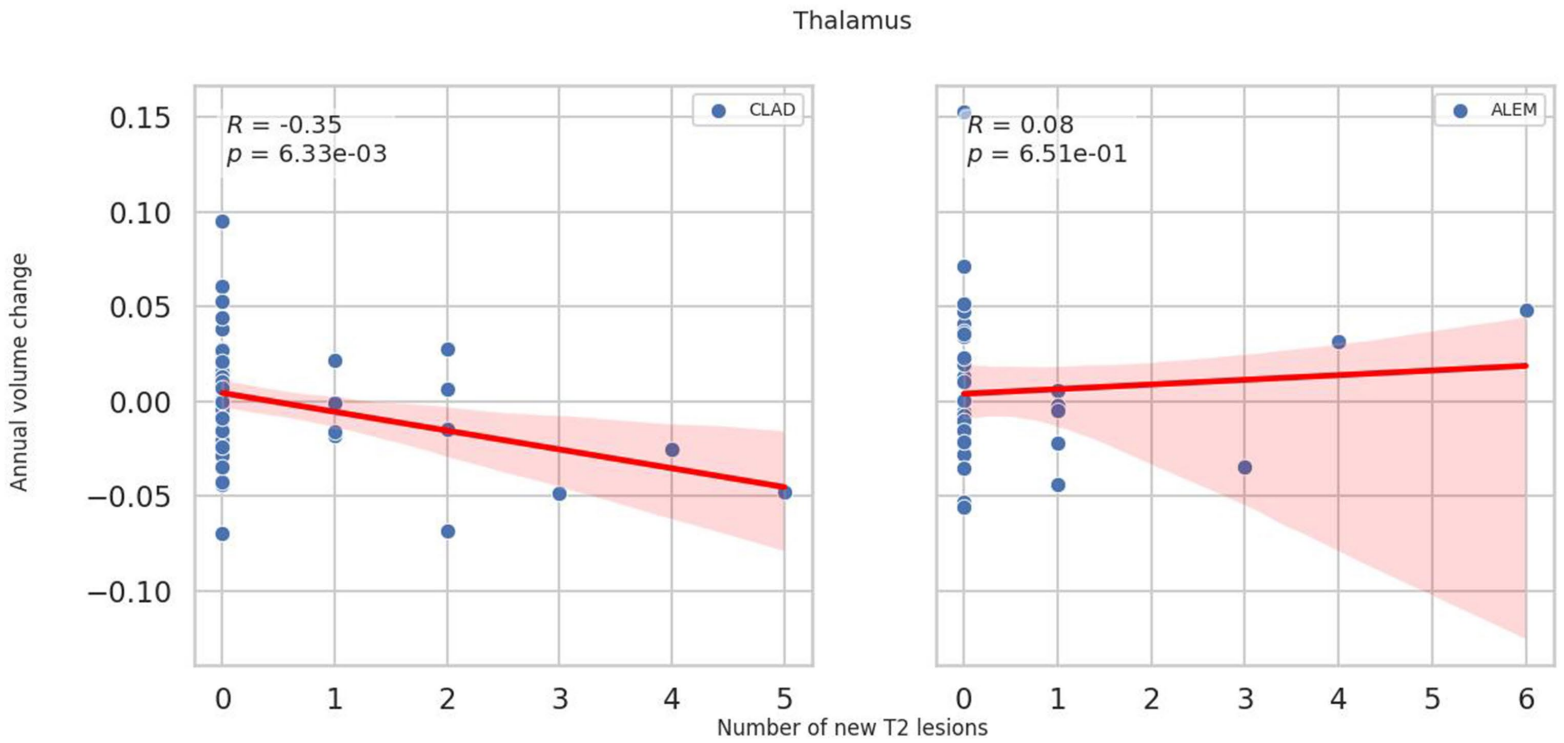
Correlation between the number of new T2 lesions and annual change in thalamus volume in the corresponding year. Each point represents a patient-year, pooled across the 3-year follow-up. A linear regression line with a 95% confidence interval is shown. The reported p-value and R^2^ refer to this simple linear regression and not to the linear mixed-effects model used in the main analysis.

In the subsequent years of follow-up, new T2 lesions were absent in most patients across both treatment groups. Nevertheless, significant group differences were present in the associations between new T2 lesions and atrophy rates for the cerebellum (*p* = 0.02; Figure 1), deep GM (DGM; *p* = 0.04; Figure 2), and thalamus (*p* = 0.04; Figure 3), as summarized in Table 2. In the CLAD group, each new T2 lesion was associated with an annual volume decrease of approximately −0.05, −0.01, and −0.01% of eTIV in the cerebellum, DGM, and thalamus, respectively. By contrast, in the ALEM group, these structures showed a non-significant trend toward volume increase.

**Table 2:** Year-to-year correlations between the number of new T2 lesions and annual brain volume changes in the corresponding years.

Structure	CLAD			ALEM			Interaction	
	Slope (*β*)	*p*-value	*n*	Slope (*β*)	*p*-value	*n*	*p*-value	*n*
Total WM	−0,31,265	0,32,629	59	0,01221	0,97,045	36	0,47,945	95
Whole brain	−0,27,485	0,09142	59	0,13,103	0,43,633	36	0,08278	95
Cerebellum	−0,05115	0,05479	59	0,03703	0,16,803	36	0,02158	95
Cortex	0,05540	0,76,623	59	0,08571	0,65,597	36	0,91,070	95
DGM	−0,01470	0,04721	59	0,00698	0,35,835	36	0,04057	95
Total GM	−0,00031	0,99,870	59	0,11,913	0,54,249	36	0,66,279	95
Thalamus	−0,00989	0,02153	59	0,00247	0,57,166	36	0,04325	95
Caudate	−0,00035	0,91,061	59	0,00080	0,80,028	36	0,79,481	95
Hippocampus	0,00132	0,53,023	59	0,00490	0,02681	36	0,21,086	95
Amygdala	−0,00253	0,05816	59	0,00065	0,61,017	36	0,08110	95
Lateral ventricle	0,03447	0,06179	59	−0.00035	0,98,547	36	0,18,813	95

Regression slopes and p-values are derived from the linear mixed-effects model and are presented separately for the CLAD and ALEM groups. The last column reports the *p*-value for the interaction term, indicating whether the strength of the association between new T2 lesions and volume change differs between the two groups. *n* = the number of patient-years; DGM, deep grey matter volume; total WM, total white matter volume; total GM, total grey matter volume. Statistically significant values are highlighted with a green background.

### 3.4 Correlation of three-year volumetric changes with baseline T2/Gd + lesions

**Figure 4:**
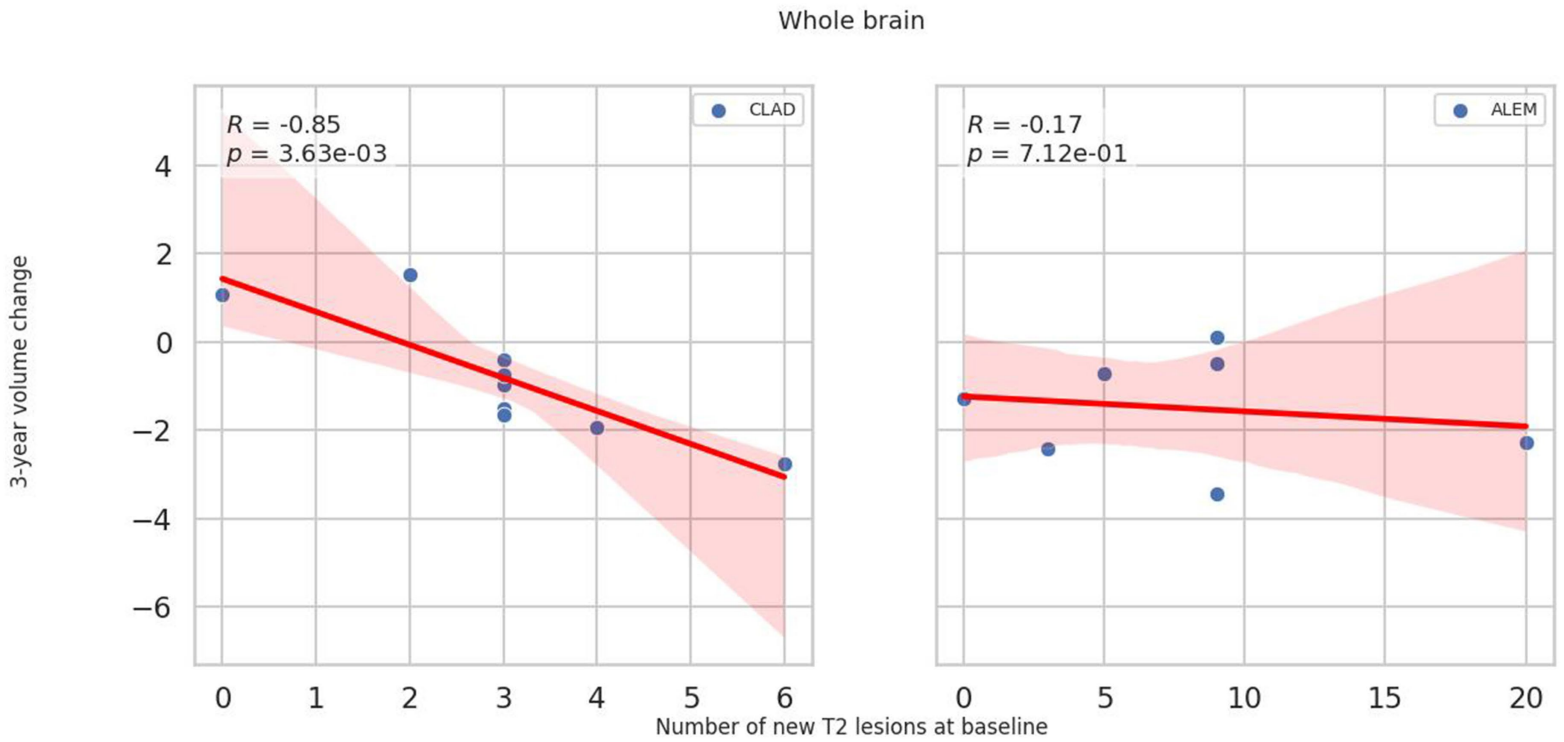
Correlation between number of T2 lesions at baseline and 3-year change of whole brain volume. A linear regression line with 95% confidence interval was shown.

**Figure 5:**
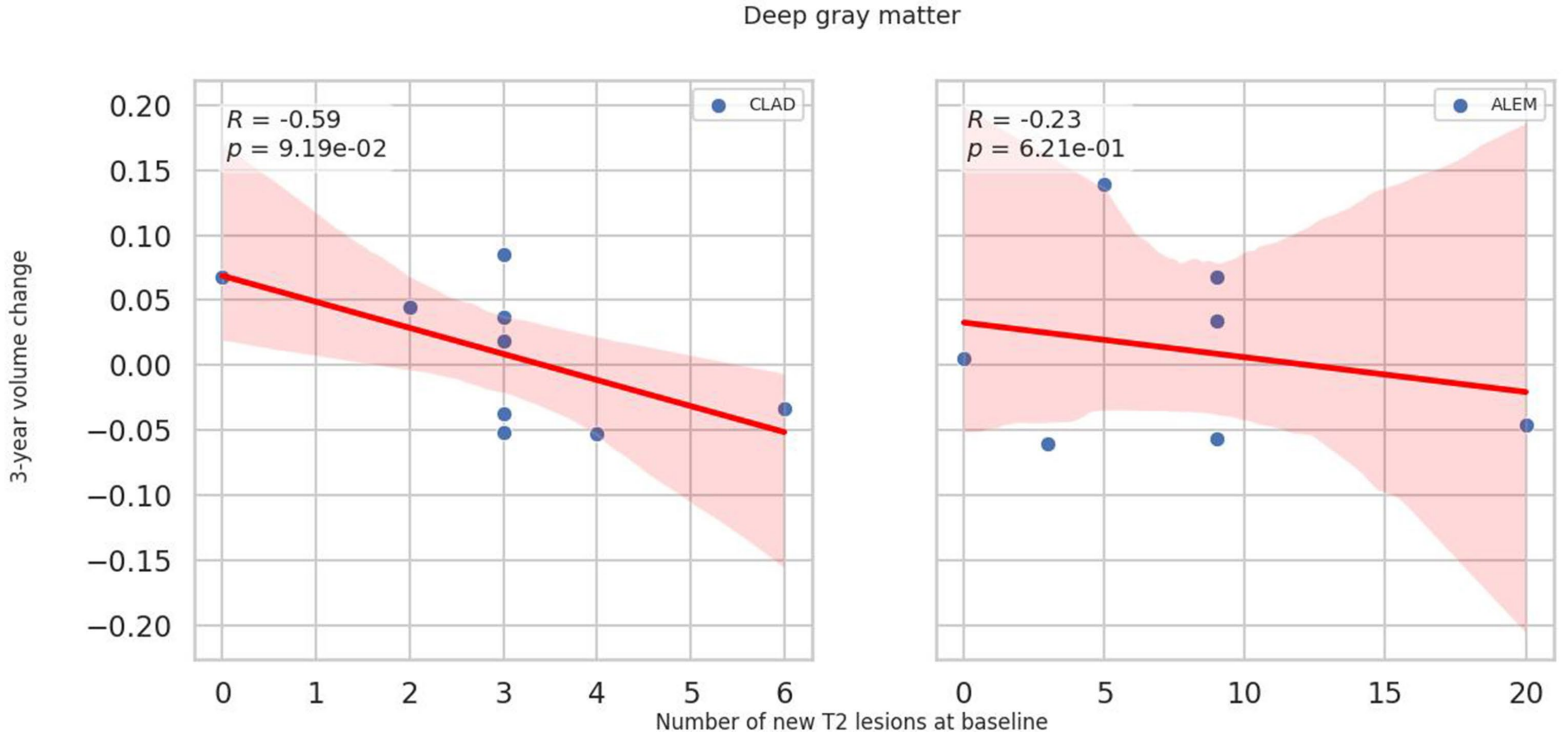
Correlation between number of T2 lesions at baseline and 3-year change of deep grey matter volume. A linear regression line with 95% confidence interval was shown.

**Figure 6:**
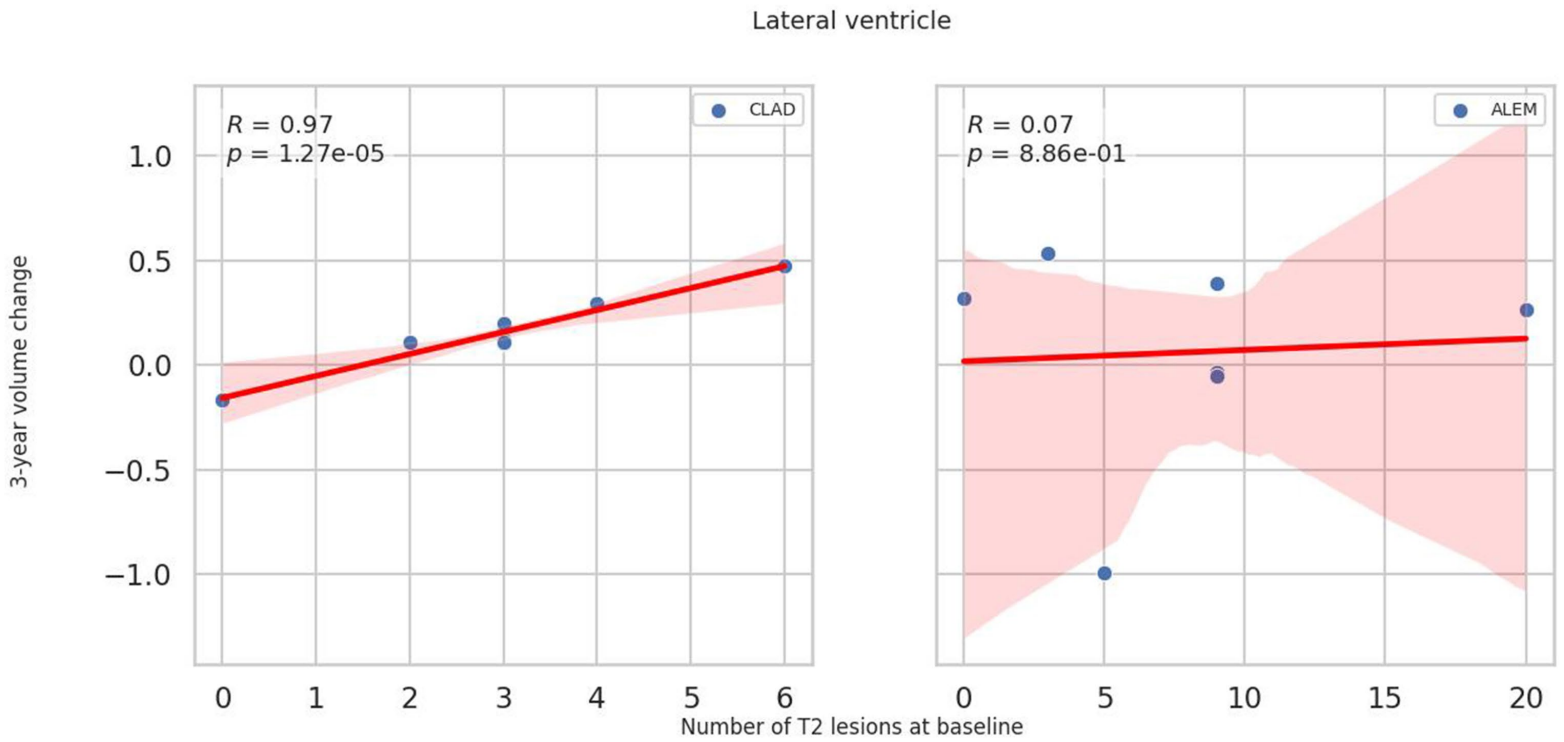
Correlation between number of T2 lesions at baseline and 3-year change of lateral ventricle volume. A linear regression line with 95% confidence interval is shown.

In relation to baseline T2 lesions, significant differences between groups were observed. In the CLAD group, the baseline number of T2 lesions statistically significantly correlated with whole brain (*p* = 0.001, *ꞵ* = −0.89; Figure 4), cerebellum (*p* = 0.002, *ꞵ* = −0.20), cerebellar cortex (*p* = 0.003, *ꞵ* = −0.19) and DGM (*p* = 0.015, *ꞵ* = –0.04; Figure 5) atrophy, as well as with lateral ventricular volume increase (*p* = 0.00001, *ꞵ* = 0.1; Figure 6; Table 3). For the ALEM group, such a relationship was not noted.

**Table 3:** Correlations between the number of new T2 lesions at baseline and three-year volume changes.

Structure	ALL				CLAD				ALEM			
	Slope (*β*)	*p*-value	*R* ^2^	*N*	Slope (*β*)	*p*-value	*R* ^2^	*N*	Slope (*β*)	*p*-value	*R* ^2^	*N*
Lateral ventricle	0.00438	0.82258	0.01	16	0.10487	0.00001	0.94	9	0.00541	0.88568	0.01	7
Thalamus	−0.00278	0.40848	0.05	16	−0.02001	0.09193	0.35	9	−0.00267	0.62083	0.05	7
Amygdala	−0.00095	0.17049	0.13	16	−0.00184	0.42036	0.10	9	−0.00002	0.97881	0.01	7
DGM	−0.01038	0.05557	0.24	16	−0.03957	0.01543	0.59	9	−0.00599	0.48624	0.10	7
Whole brain	−0.10578	0.22282	0.10	16	−0.88808	0.00136	0.79	9	−0.03463	0.77891	0.02	7
Total WM	−0.09039	0.55211	0.03	16	−0.37000	0.38031	0.11	9	0.10083	0.66677	0.04	7
Cerebellum	−0.03284	0.07939	0.20	16	−0.19689	0.00228	0.76	9	−0.02627	0.31612	0.20	7
Total GM	−0.01952	0.84888	0.01	16	−0.51992	0.07922	0.38	9	−0.13693	0.29892	0.21	7
Cerebellar cortex	−0.02680	0.11749	0.17	16	−0.18598	0.00263	0.75	9	−0.02070	0.35813	0.17	7
Caudate	−0.002721	0.57354	0.12	16	0.00347	0.50229	0.20	9	−0.00294	0.82353	0.12	7
Hippocampus	−0.001791	0.19232	0.12	16	−0.00122	0.50402	0.07	9	0.00012	0.95359	0.01	7

*p*-values, regression slopes and *R*^2^ are derived from simple linear regression analyses. Results are shown for the CLAD and ALEM groups, as well as for both groups combined (ALL). *N* = number of patients; DGM, deep grey matter volume; total WM, volume of total white matter; total GM, volume of total grey matter. The green background color was used to highlight statistically significant values.

Similar correlations were not observed for both new Gd + lesions in the year-to-year analysis and baseline Gd + lesions in the three-year analyses.

### 3.5 Correlations between baseline T2/Gd + lesions and volume changes in the first year of treatment

In the ALEM group, one patient presented with over 20 new Gd + and T2 lesions at baseline. Because this number of lesions was markedly higher than in all other participants and given the small group size (*N* = 14), this individual had a disproportionate influence on the statistical significance of the results. When this outlier was excluded, no statistically significant associations were observed within the ALEM group, and these results were reported.

In the CLAD treatment group a negative correlation between the baseline number of T2 lesions and changes in thalamus (*p* = 0.0186, *ꞵ* = –0.004), DGM (*p* = 0.0146, *ꞵ* = –0.008), total GM (*p* = 0.0360, *ꞵ* = –0.087), cerebellar cortex (*p* = 0.00001, *ꞵ* = –0.035), cerebellum (*p* = 0.0235, *ꞵ* = –0.018) and whole brain volume (*p* = 0.0372, *ꞵ* = –0.129) was noted (Supplementary Table S1). In addition, a positive correlation between the baseline number of T2 lesions and the increase in lateral ventricular volume in the first year of follow-up was reported (*p* = 0.0014, *ꞵ* = 0.019; Supplementary Table S1). Remarkably, in the ALEM group, the number of baseline T2 lesions did not influence the degree of atrophy in the first year of treatment.

No similar correlations were observed between the baseline number of Gd + lesions and volumetric changes in the first year of follow-up.

### 3.6 Comparison of years with and without relapses for all patients in terms of volumetric changes

When comparing years with and without relapses in all patients, no statistically significant differences were observed between groups in relation to brain atrophy.

### 3.7 Comparison of volumetric changes between patient-years with and without NEDA-3

**Figure 7:**
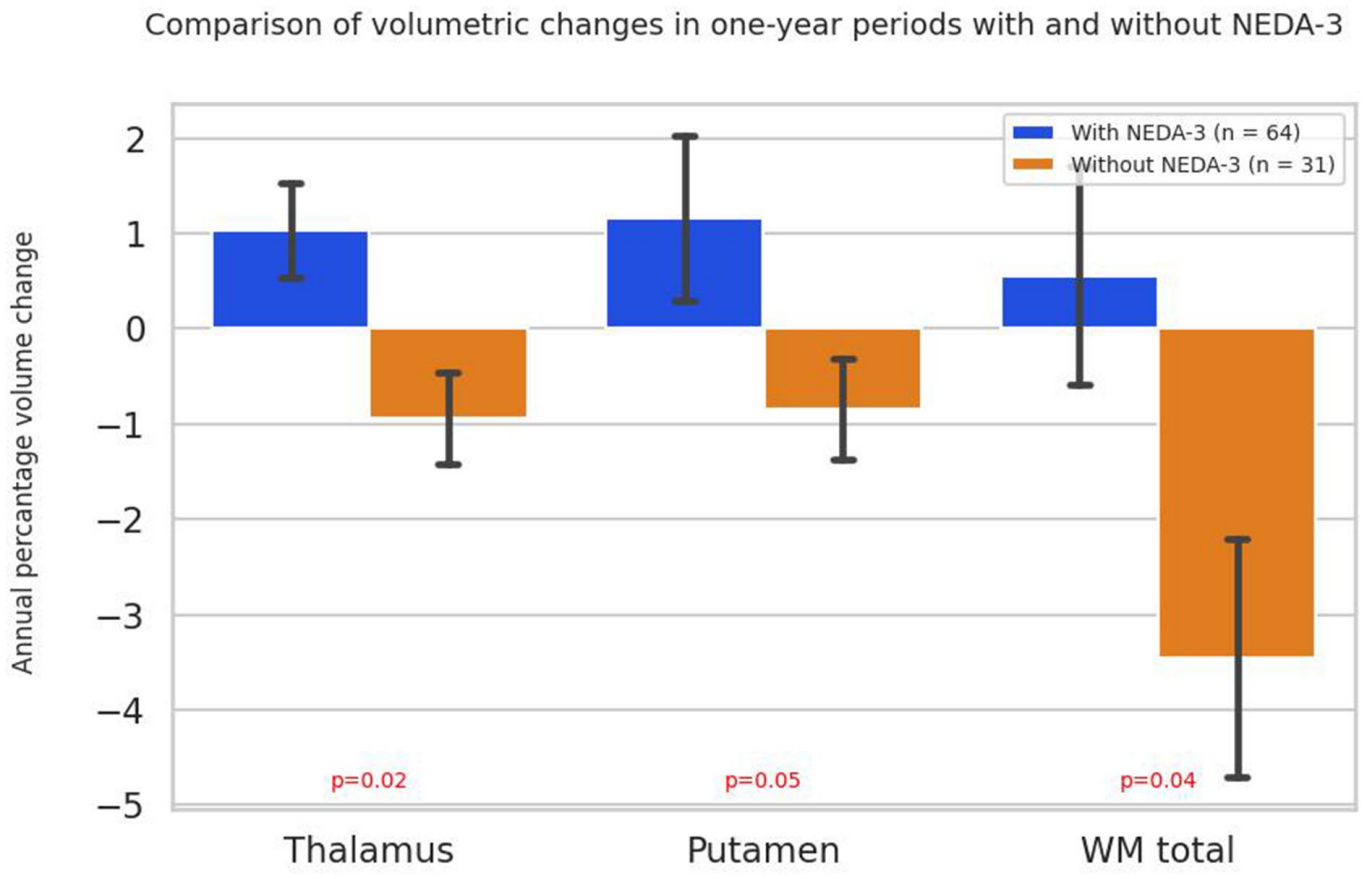
Annual percentage volume changes in the thalamus, putamen and white matter in all patients grouped by NEDA-3 criteria. Bars represent mean percentage volume change, with standard errors indicated. *p*-values were obtained using the Mann–Whitney U test. WM total - volume of total white matter.

When comparing all years of follow-up in all patients from both treatment groups who did not achieve NEDA-3 status, statistically significantly greater atrophy of WM (*p* = 0.04), thalamus (*p* = 0.02) and putamen (*p* = 0.04) was observed compared to patients achieving NEDA-3 (Figure 7).

## 4 Discussion

The aim of the presented study was to explore the possible correlation of clinical and radiological disease activity with brain atrophy in MS patients. As shown by the results of our study, volumetric changes in some brain regions correlated with radiological progression in the form of new T2 lesions as well as their baseline number, which may make them suitable markers for assessing radiological disease activity. It is worth noting that this correlation was the most visible in the CLAD-treated group, which may suggest a strong potential of ALEM to inhibit the progression of atrophy regardless of the burden of demyelinating lesions, as well as to prevent their formation. However, it should be noted here, that in both drug groups no new lesions were observed in the subsequent years of treatment in the majority of patients. This is consistent with our previous article, in which we have already described that IRT therapy seems to be promising in terms of inhibiting atrophy in patients with RRMS (Pogoda-Wesołowska et al., 2025).

As described above, in the CLAD group, the appearance of new T2 lesions correlated with atrophy of the thalamus, DGM, and cerebellum. Furthermore, a similar correlation was observed between the baseline number of T2 lesions and increased lateral ventricular volume and atrophy of the DGM, whole brain and the cerebellum after 3 years of follow-up. Such associations were not observed for Gd + lesions. This may suggest that brain volume loss is significantly related to T2 lesions volume but partially independent of lesion inflammatory activity, which may explain the limited efficacy of anti-inflammatory treatment on the neurodegenerative process.

In relation to research conducted so far, Miller et al. showed that T2 lesion volume at baseline was strongly associated with subsequent brain volume loss (*p* < 0.0001; Miller et al., 2018). Moreover, in their study, the presence of Gd + lesions at baseline had a weaker but noticeable correlation with brain atrophy. Also, in the study by Radue et al., T2 lesions volume and number of Gd + lesions at baseline were the strongest predictors of brain volume loss over 2 years (Radue et al., 2015). Patients with a higher T2 lesions burden and more Gd + lesions experienced greater brain volume loss. In addition, in the study by De Stefano et al., in MS patients treated with fingolimod, T2 and Gd + lesions burden at baseline were strong predictors of brain volume loss over 2 years (De Stefano et al., 2017). Interestingly, the study by Siger showed that patients with primary progressive MS (PPMS) had larger T2 and Gd + lesions volumes and faster brain volume loss compared with patients with RRMS (Siger, 2022). Furthermore, the presence of Gd + lesions at disease onset was associated with a faster rate of brain atrophy. In the study by Chard et al., T2 lesion volume was negatively correlated with loss of brain parenchyma fraction (BPF; *p* < 0.001) and GM fraction (GMF; *p* < 0.001) but not with WM fraction (WMF; *p* = 0.134). Gd + lesions volumes were not correlated with either volume. The lack of correlation between lesion burden measures and WMF suggests that pathological changes in WM may occur by mechanisms that are at least partially independent of the overt genesis of lesions in the early phase of MS (Chard et al., 2002). Remarkably, in the study by Genovese et al., in a 10-year follow-up atrophied T2 lesions (an imaging measure that reflects the replacement of T2 lesions by cerebrospinal fluid spaces) volume was a robust MRI marker of MS disability progression and conversion into a secondary progressive disease course (SPMS; Genovese et al., 2019). Similarly, in the study of Zivadinov et al., the volume of T2 lesions strongly correlated with progression to the secondary progressive phase and disability (Zivadinov et al., 2019). Moreover, in the study of Tavazzi et al. in 127 patients (RRMS/PPMS), the volume of T2 lesions was the only marker differentiating disease progression (*p* = 0.007; Tavazzi et al., 2020). The volume of T2 lesions was shown to be associated with the total brain volume, thalamus volume and cognitive test results. Interestingly, in their other study baseline sNfL level, T2 lesions volume, and total brain atrophy were strongly correlated (Tavazzi et al., 2019). However, only T2 lesions volume significantly predicted disease progression (*p* = 0.006). Interestingly, in a study conducted by Oship et al. enlargement of T2 lesions was more strongly associated with long-term disability progression compared to other conventional T2 lesion-based outcomes (Oship et al., 2022).

Another important observation in the presented study was the fact that when comparing patients with relapses with those who did not experience a relapse in all years of follow-up, no statistically significant differences were observed in relation to volumetric changes. However, in patients who achieved NEDA-3 status, a statistically significant slowing of thalamic, WM and putamen atrophy was noted compared to patients without NEDA-3. This was probably related to the greater impact of radiological disease progression on brain atrophy compared to clinical activity.

These findings correspond with the study by Akaishi et al., which showed that patients without relapses had a similar rate of brain atrophy to patients with relapses, suggesting that brain atrophy may progress independently of relapse activity (Akaishi et al., 2024). In contrast, in the work of Cagol et al., the authors observed that among individuals who relapsed without progression independent of relapse activity (PIRA) events, there were increased rates of atrophy of total brain volume (*p* = 0.04) and total GM (*p* = 0.04), which were evident in both cortical GM (*p* = 0.04) and DGM (*p* = 0.04; Cagol et al., 2022). Furthermore, accelerated thinning of the entire cerebral cortex (*p* = 0.04) as well as temporal, parietal, occipital, insular, and cingulate cortices was noted in these patients. Interestingly, in the work of Temmerman et al., the annual percentage change in whole brain volume was similar in 29 MS patients who achieved NEDA-3 and 24 healthy controls (*p* = 0.9992; Temmerman et al., 2022). In opposite, in the second group of MS patients (*n* = 21), who were excluded from the NEDA-3 group due to disease activity (*p* = 0.1371), a mean increase in BVL of 72% was observed compared to the first group. It suggested that patients achieving NEDA-3 status have a rate of brain volume loss comparable to healthy individuals, whereas patients with disease activity lose brain volume much faster. However, in the study by Yokote et al., which included 22 Japanese MS patients, nine (64.3%) patients with NEDA-3 had significant BVL, defined as ≥ 0.4% per year (Yokote et al., 2018). Importantly, these nine patients included all patients receiving interferon-*β* (INFB) therapy (*n* = 6). While patients treated with fingolimod after IFN-β did not have significant BVL. These results suggested that NEDA-4 assessment, especially in patients treated with interferon-β, was recommended in MS clinical practice in Japan, although Japanese MS patients are generally considered to have a milder disease course, including brain atrophy, compared with their Western counterparts. Remarkably, in the study by Hanninen et al., in which total and regional brain volumes were measured in 24 patients with RRMS and 36 patients with SPMS after 6 months of treatment and after 2 years of follow-up, only 2/16 patients with isolated thalamic atrophy (*p* = 0.012) achieved NEDA-3 status (Hänninen et al., 2019). Patients with isolated thalamic atrophy had a higher risk of not achieving 2-year NEDA-3 than patients without identified brain atrophy. The groups were clinically indistinguishable. This suggested that a single measurement of thalamic and whole-brain atrophy could help identify patients in need of the most effective therapies from the outset. This was consistent with our results, in which patients not achieving NEDA-3 status had statistically significantly greater thalamic atrophy compared with those with NEDA-3.

The presented study had several limitations, with the small patient cohorts being the most important one. MRI scans conducted within 8 weeks of intravenous steroid administration were excluded from the analysis to minimize the risk of pseudoatrophy. Additionally, MRI examinations that did not meet quality standards due to artifacts or protocol inconsistencies were also excluded, resulting in missing imaging data for some patients. Although all these limitations reduced the size of the analyzed group and increased the risk of false positives, they also strengthened the reliability of the retained results by minimizing potential artifacts and confounding effects. The retrospective design inherently limits the ability to fully control for potential confounding factors and introduces the risk of bias. Despite the exclusion of patients treated with steroids in a short interval from the study, the influence of previous therapies on the results cannot be excluded. In addition, patients received different numbers and types of DMTs, which may affect the degree of atrophy irrespective of the treatment under investigation. Moreover, only T1-weighted images were used for volumetric analysis due to the application of the standard Free Surfer pipeline, without the inclusion of additional FLAIR sequences. Furthermore, the T1 images were not subjected to advanced pre-processing steps, such as lesion filling, which could impact the accuracy of atrophy measurements. An additional limitation relates to the statistical approach. Multiple comparisons correction was not applied. It should be emphasized here that this was an exploratory, hypothesis-generating study, and thus intentionally refrained from overinterpreting results. The exploratory design of this study therefore provides preliminary insights, but not confirmatory evidence. Therefore, further studies involving larger patient cohorts are necessary to more accurately characterize the relationship between brain atrophy patterns and disease activity.

In conclusion, the presented study suggested the role of demyelinating lesions (T2) in predicting atrophy of some brain areas, such as the thalamus or DGM. It also indicated a smaller association of active inflammation (Gd + lesions) with brain volume loss. Moreover, it demonstrated that neurodegeneration in MS is more likely to be due to radiological progression of the disease than clinical activity. Finally, it pointed to the role of certain brain regions and their volume changes over time in predicting further disease activity and progression. The observed differences between the ALEM and CLAD groups should be interpreted with caution. They may represent a true signal reflecting differences in the biological effects of the two treatments and, as mentioned above, suggest a strong neuroprotective potential for ALEM therapy. However, studies comparing these two drugs, particularly in the context of RWE and inhibition of atrophy progression, are lacking to confirm this hypothesis. In study by Bose et al. ALEM and CLAD achieved similar rates of NEDA in long-term follow-up, with overall less adverse events with cladribine (Bose et al., 2021). Whereas Roos et al. demonstrated that both the mean ARR and the cumulative risk of relapse were lower in patients treated with ALEM than with CLAD (Roos et al., 2024). However, apart from our own study, the authors of this article did not find any work comparing the effect of IRT on the degree and pattern of brain atrophy (Pogoda-Wesołowska et al., 2025). Therefore, as mentioned above, due to the exploratory, hypothesis-generating nature of the study, its retrospective design, and the small number of treatment groups, the conducted analysis should be considered a pilot study. Hence, further prospective studies with strictly defined inclusion and exclusion criteria, conducted on larger groups of patients, are necessary to confirm the presented results.

## Data Availability

The original contributions presented in the study are included in the article/Supplementary material, further inquiries can be directed to the corresponding author.
