# Supplementary material for: Assessment of brain atrophy as a promising marker of radiological activity in patients with relapsing–remitting multiple sclerosis
Source: Front Neurosci. 2025 Oct 7;19:1661539. doi: 10.3389/fnins.2025.1661539 (PMC12537736; doi:10.3389/fnins.2025.1661539)
Supplement: Supplementary file 1 [file Table_1.docx]

***Supplementary Material***

|  | **ALL** | | | | **CLAD** | | | | **ALEM** | | | |
| --- | --- | --- | --- | --- | --- | --- | --- | --- | --- | --- | --- | --- |
|  | **slope** | **p_value** | **R^2^** | **N** | **slope** | **p_value** | **R^2^** | **N** | **slope** | **p_value** | **R^2^** | **N** |
| **Lateral ventricle** | 0.00969 | 0.02147 | 0,13 | 42 | 0.01920 | 0.00141 | 0,32 | 29 | -0.00407 | 0.42350 | 0,07 | 13 |
| **Thalamus** | -0.00219 | 0.09928 | 0,07 | 42 | -0.00447 | 0.01859 | 0,19 | 29 | 0.00165 | 0.33789 | 0,09 | 13 |
| **Amygdala** | -0.00041 | 0.28679 | 0,03 | 42 | 0.00003 | 0.94391 | 0,01 | 29 | -0.00057 | 0.50463 | 0,05 | 13 |
| **DGM** | -0.00687 | 0.00380 | 0,20 | 42 | -0.00774 | 0.01463 | 0,20 | 29 | -0.00291 | 0.43601 | 0,06 | 13 |
| **Whole brain** | -0.08264 | 0.04071 | 0,10 | 42 | -0.12873 | 0.03722 | 0,15 | 29 | -0.03440 | 0.39832 | 0,07 | 13 |
| **Total WM** | -0.00416 | 0.93102 | 0,01 | 42 | -0.04195 | 0.54644 | 0,01 | 29 | 0.07102 | 0.30653 | 0,10 | 13 |
| **Cerebellum** | -0.01149 | 0.08171 | 0,08 | 42 | -0.01828 | 0.02353 | 0,18 | 29 | 0.00138 | 0.92014 | 0,01 | 13 |
| **Total GM** | -0.07956 | 0.02057 | 0,13 | 42 | -0.08731 | 0.03598 | 0,15 | 29 | -0.10519 | 0.15257 | 0,19 | 13 |
| **Cerebellar cortex** | -0.01905 | 0.00213 | 0,22 | 42 | -0.03473 | 0.00001 | 0,53 | 29 | 0.00351 | 0.74546 | 0,01 | 13 |
| **Caudate** | -0.00102 | 0.21381 | 0,03 | 42 | -0.00159 | 0.19532 | 0,06 | 29 | 0.00018 | 0.85523 | 0,01 | 13 |
| **Hippocampus** | -0.00187 | 0.00468 | 0,19 | 42 | -0.00107 | 0.14981 | 0,08 | 29 | -0.00156 | 0.14547 | 0,20 | 13 |

**Supplementary Table S1.** Correlations between the number of new T2 lesions at baseline and first-year volume changes. P-values, regression slopes and R^2^ are derived from simple linear regression analyses. Results are shown for the CLAD and ALEM groups, as well as for both groups combined (ALL). DGM – deep grey matter volume, total WM – volume of total white matter, total GM – volume of total grey matter. N=number of patients.
